# Effects of environmental, social and surgical factors on ovarian reserve: Implications for age‐relative female fertility

**DOI:** 10.1002/ijgo.13567

**Published:** 2021-02-11

**Authors:** Ying Wang, Yuncang Yuan, Dan Meng, Xiaona Liu, Yucui Gao, Fang Wang, Yuyan Li, Wei He

**Affiliations:** ^1^ Reproductive Medical Center Department of Obstetrics and Gynecology Southwest Hospital Third Military Medical University (Army Medical University Chongqing China

**Keywords:** anti‐Müllerian hormone, antral follicle counts, female fertility, risk factor

## Abstract

**Objective:**

To investigate new risk factors for female fertility by analyzing the effects of environmental, social, and surgical factors on antral follicle counts (AFC) and anti‐Müllerian hormone (AMH) levels.

**Methods:**

A total of 1513 women aged 20–47 years who underwent in vitro fertilization/intracytoplasmic injection treatment in Southwest Hospital from December 2017 to December 2019 were included. Women were assessed for AFC and AMH levels, and completed a questionnaire. Ordinal logistic regression analyses with generalized linear mixed models were used to calculate the adjusted odds ratio (OR) for diminished ovarian reserve.

**Results:**

Adnexal surgery was the only risk factor associated with low AFC in women aged 20–30 years. Younger age at menarche, alcohol drinking, and adnexal surgery are three independent risk factors for AMH decline in women aged 20–30 years. Intense exercise, sleep quality, and adnexal surgery are three independent risk factors for a low AFC in women aged 31–36 years. Alcohol drinking and adnexal surgery are two independent risk factors for AMH decline in women aged 31–36 years.

**Conclusion:**

With age, female fertility becomes sensitive to high‐intensity exercise and poor sleep quality. Adnexal surgery and alcohol drinking are two important risk factors for female fertility in women under age 37 years.

## Synopsis

This study demonstrated the negative effects of high‐intensity exercise, poor sleep quality, adnexal surgery, and alcohol drinking on fertility in age‐relative women.

## 1 INTRODUCTION

Reproduction is one of the most conserved phenomena during species evolution. Surveys from the past two decades have shown that infertility has increased in relatively younger women. Industrialization, environmental pollution, living pressures, and illnesses are negative factors affecting human fertility.

The ovarian reserve is a common indicator for female fertility.^1^ Age, antral follicle count (AFC), anti‐Müllerian hormone (AMH) level and basal sex hormone level, including follicle‐stimulating hormone, luteinizing hormone, and estradiol, are typically used to determine the relative condition of the ovarian reserve in clinical practice, especially the combinations of age, AFC, and AMH.^2^

A number of factors affecting AFC or AMH levels have been reported, some are considered controversial or have been reported in limited studies. A committee opinion indicated that smoking has some potential deleterious effects on female fertility by delaying conception, impairing ovarian follicular dynamics, inducing gamete mutations, and increasing the risk of early miscarriages and adverse assisted reproductive technology outcomes.^3^ Alcohol was considered to be a risk factor of female fertility through altering endogenous hormone concentrations, hindering ovum maturation, and disturbing ovulation, early blastocyst development, and implantation.^4^ The relationships between smoking, alcohol and AFC or AMH levels are ambiguous. Peck et al.^5^ provided the first morphologic evidence that smoking decreased human ovarian follicle number, but subsequent studies reported no such association.^6^ Similarly, several studies have demonstrated that alcohol and smoking are not related to AMH levels,^6^, ^7^, ^8^ but other studies have reported negative associations.^9^, ^10^ The effects of age at menarche on AMH levels remains controversial^6^, ^8^; and associations among additional factors, such as pesticides^9^—which may disturb the hormonal function,^11^ and drinking coffee,^7^ and AFC or AMH levels have been analyzed in limited studies.

In our previous study, we preliminarily analyzed the risk factors for female fertility in 794 women aged under 37 years and found that adnexal surgery, which may disturb the blood supply of female ovaries and impair the normal microenvironment of antral follicle development, was associated with decreased AFC and AMH levels. We found no associations among intense exercise and AFC, body mass index (BMI, calculated as weight in kilograms divided by the square of height in meters) and AMH levels, which was not consistent with previous studies.^10^, ^12^ The present study analyzed the risk factors affecting female fertility with an expanded sample size and with more age groups. This work will provide more reliable evidence for female fertility protection for different female age populations.

## 2 MATERIALS AND METHODS

Using a cross‐sectional study design, we analyzed the clinical date of 1513 women aged 20–47 years who underwent in vitro fertilization/intracytoplasmic sperm injection treatment in Southwest Hospital from December 2017 to December 2019. To minimize noise, women with chromosomal abnormalities, reproductive tract infections, or surgical history outside the pelvic cavity were excluded. Baseline information was collected, including age, BMI, and AFC and AMH levels. The study was approved by the Medical Ethics Committee of the Army Military Medical University (ethics number: AF/SC‐08/1.0). Written informed consent was waived due to the retrospective nature, and the patient data were used anonymously.

A questionnaire was designed based on the literature^13^, ^14^, ^15^ and included basic information (e.g., demographic data, medical history, reproductive history, and physiologic cycle), living habits and environmental contact (e.g., exercise, smoking and drinking, sleep and rest, and pesticide and organic contact), and dietary habits and emotional states (e.g., liquid intake, food intake, dietary supplement intake, and work stress). Specifically, alcohol drinking referred to whether or not women had consumed alcohol in the past half year, smoking referred to whether or not women had consumed at least one cigarette per day in the past half year, and sleep quality referred to whether or not women had good sleep quality in the past month. Representative variables associated with AFC and AMH levels were selected from the questionnaire based on interests, the availability of data, and previous studies.^9^, ^10^

Two stimulation protocols were used in this study, the gonadotropin‐releasing hormone long agonist protocol and the gonadotropin‐releasing hormone antagonist protocol. The total number of 2‐ to 9‐mm follicles in both ovaries in the early follicular phase was counted as AFC using ultrasound guidance by the same experienced gynecologist. Blood samples were collected on a random day of the menstrual cycle, and serum AMH was measured with an AMH ELISA kit (Kangrun Biotech) using an Access II Immunoassay System (Beckman Coulter) by the same experienced laboratory technician. The standard range of detection for the AMH assay was 0.06–18 ng/ml. Double cut‐off values of AFC for predicting ovarian reserve were defined as five and ten (bilateral ovaries) based on previous reports.^16^, ^17^, ^18^ Double cut‐off values of AMH levels for predicting ovarian reserve were 1.87 and 5.22 ng/ml according to the thresholds used for diagnosis of infertility at our center.^19^ Detailed categorizations were as follows: women with AFC ≤ 5 or AMH ≤ 1.87 ng/ml have poor ovarian reserve; women with 5<AFC<10 or 1.87<AMH<5.22 ng/ml have relatively good ovarian reserve; women with AFC ≥ 10 or AMH ≥ 5.22 ng/ml have good ovarian reserve.

Non‐normally distributed data, including age, BMI, AFC, and AMH, were presented as medians or centiles. Frequencies were compared using the χ^2^ test or Kruskal–Wallis *H* test. Univariate and multivariate ordinal logistic regression analyses in the generalized linear mixed models were used to assess the relationship between candidate variables and AFC or AMH by calculating odds ratio (OR) values. For continuous predictors, OR > 1 indicates that poor ovarian reserve and relatively good ovarian reserve are more likely as the predictor increases, and OR < 1 indicates that good ovarian reserve and relatively good ovarian reserve are more likely as the predictor increases. For categorical predictors, OR > 1 indicates that poor ovarian reserve and relatively good ovarian reserve are more likely at the level of the predictor than at the reference level of the predictor, and OR < 1 indicates that good ovarian reserve and relatively good ovarian reserve are more likely at the level of the predictor than at the reference level of the predictor. Data were analyzed using SPSS version 25.0 software (IBM Corp., Armonk, NY, USA). A *P* value less than 0.05 was considered significant.

## 3 RESULTS

**FIGURE 1:**
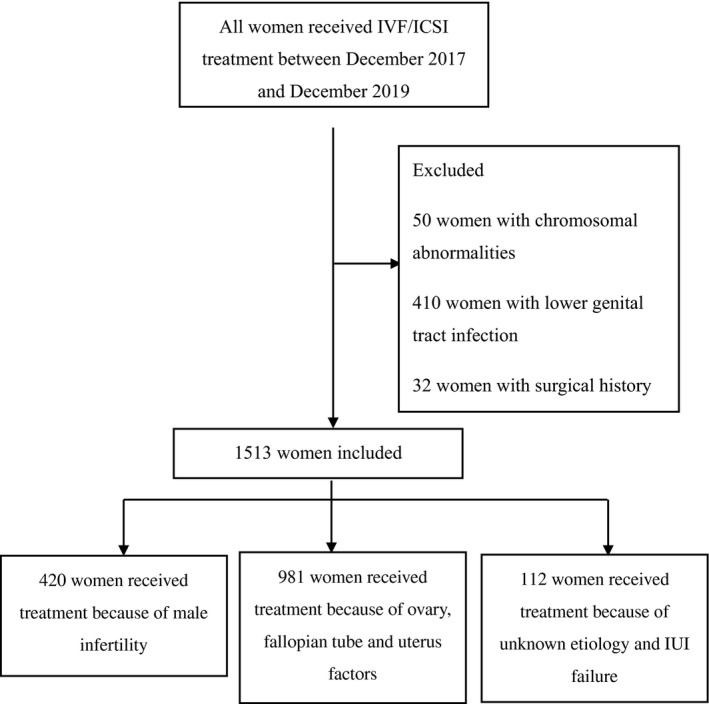
Flowchart of participants

Figure 1 shows the process of women's selection to achieve the final study sample. To minimize the disturbance, 410 women with lower genital tract infection and 32 women with surgical history outside the pelvic cavity were excluded. A further 527 women with uncompleted questionnaires were also excluded. Finally, the study sample comprised 1513 women of whom 420 were undergoing treatment because of the male factor infertility, 981 were because of the issues concerning the ovary, fallopian tube, and uterus, and 112 because of unknown etiology and intrauterine insemination failure. The median age was 30 years (interquartile range [IQR] 28–34 years), and the median BMI was 21.8 (IQR 20–24). The median AFC value was 13 (IQR 9–19) and the median AMH level was 3.28 ng/ml (IQR 1.73–5.92 ng/ml). Patients were stratified into three age groups (20–30, 31–36; ≥37 years): the median AMH levels were 4.11 ng/ml (IQR 2.36–6.97 ng/ml), 2.96 ng/ml (IQR 1.67–5.54 ng/ml), and 1.28 ng/ml (IQR 0.42–2.7 ng/ml), and the median AFC were 15 (IQR 11–21), 12 (IQR 8–17), and 6 (IQR 4–10), respectively. There was a remarkable positive relation between AFC and AMH (*r* = 0.7, *p *< 0.01), which was similar to the findings of a previous report.^20^ The constituent ratio of patients by age and subdivided by AFC, AMH, and 17 candidate risk factors are displayed in the Supplementary material (Tables S1 and S2).

All selected factors in this study passed the test of parallel lines (*p *> 0.05). The results of the AFC univariate analyses are shown in Table 1. These results suggested that a history of adnexal surgery (OR 1.60, 95% CI 1.09–2.35, *P* = 0.016) was associated with decreased AFC in women aged 20–30 years; high‐intensity exercise (OR 1.60, 95% CI 1.08–2.36, *P* = 0.018), poor quality sleep (OR 1.38, 95% CI 1.08–1.77, *p *= 0.011), and history of adnexal surgery (OR 1.77, 95% CI 1.25–2.52, *p *= 0.001) were associated with decreased AFC in women aged 31–36 years; spontaneous abortion history (OR 0.56, 95% CI 0.31–1.00, *p *= 0.050) and moving or renovating (OR 0.41, 95% CI 0.18–0.95, *p *= 0.038) seemed to be associated with increased AFC in women aged 31–36 years; and no significant association was found between any terms and AFC in women over the age of 37 years. The results of the univariate analyses for AMH are shown in Table 2. These results indicated that an early age at menarche (OR 1.20, 95% CI 1.35–1.07, *p *= 0.001), alcohol drinking (OR 1.52, 95% CI 1.07–2.15, *p *= 0.018) and a history of adnexal surgery (OR 1.45, 95% CI 1.10–1.92, *p *= 0.008) were associated with decreased AMH in women aged 20–30 years; alcohol drinking (OR 1.50, 95% CI 1.01–2.24, *p *= 0.045), and adnexal surgery (OR 1.39, 95% CI 1.02–1.91, *p *= 0.040) were associated with decreased AMH in women aged 31–36 years; and no significant associations were found between any factors and AMH levels in women over 37 years of age.

**TABLE 1:** Univariable logistic regression analysis of the risk factors associated with antral follicle count

Risk factors	≤30 years		31–36 years		≥37 years	
	OR (95% CI)	*P* value	OR (95% CI)	*P* value	OR (95% CI)	*P* value
Basic characteristics						
BMI						
>24	0.61 (0.37–1.01)	0.057	1.17 (0.79–1.73)	0.426	1.01 (0.57–1.80)	0.964
≤24	1	–	1	–	1	–
Reproduction endocrine history						
Menarche age	3.45 (0.91–1.37)	0.290	1.07 (0.93–1.23)	0.347	3.45 (0.91–1.37)	0.290
Menstrual blood volume	0.85 (0.59–1.21)	0.362	1.23 (0.91–1.68)	0.180	1.11 (0.72–1.72)	0.631
Dysmenorrhea	1.06 (0.76–1.48)	0.735	1.10 (0.83–1.45)	0.515	0.67 (0.43–1.03)	0.065
Spontaneous abortion						
Yes	0.84 (0.43–1.65)	0.618	0.56 (0.31–1.00)	0.050	0.63 (0.31–1.30)	0.212
No	1	–	1	–	1	–
Environment						
Moving or renovating						
Yes	0.41 (0.16–1.05)	0.062	0.41 (0.18–0.95)	0.038	1.49 (0.49–4.56)	0.487
No	1	–	1	–	1	–
Lifestyle						
Intense exercise						
Yes	1.00 (0.63–1.59)	0.996	1.60 (1.08–2.36)	0.018	1.17 (0.66–2.07)	0.599
No	1	–	1	–	1	–
Sleep quality	1.10 (0.82–1.47)	0.519	1.38 (1.08–1.77)	0.011	1.12 (0.74–1.68)	0.597
Tobacco smoking						
Yes	1.28 (0.54–3.02)	0.574	1.49 (0.75–2.95)	0.252	0.56 (0.16–1.95)	0.364
No	1	–	1	–	1	–
Alcohol drinking						
Yes	0.91 (0.55–1.52)	0.714	1.40 (0.91–2.16)	0.126	0.96 (0.47–1.96)	0.903
No	1	–	1	–	1	–
Tea consumption						
Yes	1.24 (0.82–1.86)	0.312	0.94 (0.66–1.35)	0.740	0.99 (0.57–1.71)	0.959
No	1	–	1	–	1	–
Coffee consumption						
Yes	1.05 (0.60–1.83)	0.873	0.89 (0.57–1.39)	0.607	0.76 (0.38–1.53)	0.440
No	1	–	1	–	1	–
Food intake						
Spicy food, times/week	0.99 (0.83–1.18)	0.927	0.95 (0.81–1.12)	0.539	0.96 (0.73–1.27)	0.792
Pickled foods	1.16 (0.77–1.75)	0.473	0.93 (0.63–1.36)	0.699	0.67 (0.39–1.16)	0.152
Smoked foods	0.79 (0.54–1.15)	0.222	0.82 (0.59–1.16)	0.266	1.33 (0.80–2.20)	0.275
Surgical factors						
Adnexal surgery						
Yes	1.60 (1.09–2.35)	0.016	1.77 (1.25–2.52)	0.001	0.79 (0.46–1.35)	0.392
No	1	–	1	–	1	–
Uterine surgery						
Yes	0.81 (0.42–1.58)	0.538	1.09 (0.70–1.70)	0.715	0.84 (0.44–1.60)	0.597
No	1	–	1	–	1	–

Abbreviations: BMI, body mass index (calculated as weight in kilograms divided by the square of height in meters);CI, confidence interval; OR, odds ratio.

**TABLE 2:** Univariable logistic regression analysis of the risk factors associated with anti‐Müllerian hormone

Risk factors	≤30 years		31–36 years		≥37 years	
	OR (95% CI)	*P* value	OR (95% CI)	*P* value	OR (95% CI)	*P* value
Basic characteristics						
BMI						
>24	0.81 (0.59–1.11)	0.195	1.22 (0.85–1.73)	0.279	0.79 (0.42–1.48)	0.452
≤24	1	–	1	–	1	–
Reproduction endocrine history						
Menarche age	1.20 (1.07–1.38)	0.001	0.98 (0.87–1.11)	0.782	1.12 (0.90–1.40)	0.315
Menstrual blood volume	0.81 (0.63–1.04)	0.100	0.95 (0.72–1.24)	0.683	1.05 (0.65–1.69)	0.859
Dysmenorrhea	1.22 (0.96–1.55)	0.101	1.00 (0.78–1.28)	0.965	0.80 (0.50–1.27)	0.337
Spontaneous abortion						
Yes	1.37 (0.87–2.16)	0.169	1.43 (0.89–2.28)	0.138	1.23 (0.56–2.68)	0.609
No	1	–	1	–	1	–
Environment						
Moving or renovating						
Yes	0.64 (0.39–1.06)	0.082	0.76 (0.41–1.39)	0.368	0.96 (0.29–3.15)	0.944
No	1	–	1	–	1	–
Lifestyle						
Intense exercise						
Yes	0.90 (0.65–1.25)	0.530	1.27 (0.89–1.82)	0.196	1.69 (0.87–3.28)	0.122
No	1	–	1	–	1	–
Sleep quality	1.07 (0.87–1.32)	0.504	1.12 (0.90–1.39)	0.334	1.54 (0.97–2.44)	0.068
Tobacco smoking						
Yes	1.05 (0.55–1.98)	0.890	1.81 (0.94–3.47)	0.075	0.60 (0.16–2.22)	0.441
No	1	–	1	–	1	–
Alcohol drinking						
Yes	1.52 (1.07–2.15)	0.018	1.50 (1.01–2.24)	0.045	0.89 (0.41–1.95)	0.766
No	1	–	1	–	1	–
Tea consumption						
Yes	1.02 (0.76–1.36)	0.910	1.23 (0.89–1.70)	0.202	1.15 (0.62–2.12)	0.665
No	1	–	1	–	1	–
Coffee consumption						
Yes	0.86 (0.58–1.27)	0.444	0.84 (0.57–1.25)	0.400	1.08 (0.49–2.35)	0.854
No	1	–	1	–	1	–
Food intake						
Spicy food, times/week	0.98 (0.86–1.11)	0.729	0.91 (0.79–1.06)	0.221	1.11 (0.82–1.51)	0.514
Pickled foods	1.24 (0.93–1.66)	0.135	1.16 (0.82–1.63)	0.403	1.12 (0.62–2.02)	0.719
Smoked foods	1.16 (0.89–1.52)	0.268	1.02 (0.75–1.38)	0.909	1.57 (0.89–2.74)	0.117
Surgical factors						
Adnexal surgery						
Yes	1.45 (1.10–1.92)	0.008	1.39 (1.02–1.91)	0.040	0.90 (0.50–1.62)	0.723
No	1	–	1	–	1	–
Uterine surgery						
Yes	1.11 (0.72–1.72)	0.641	1.04 (0.70–1.55)	0.849	1.21 (0.58–2.50)	0.611
No	1	–	1	–	1	–

Abbreviations: BMI, body mass index (calculated as weight in kilograms divided by the square of height in meters);CI, confidence interval; OR, odds ratio.

All variables with *P* < 0.05 in Tables 1 and 2 were chosen for multivariate regression analysis. The results of the multivariate analyses for AFC are detailed in Table 3. After adjusting for other confounding factors, intense exercise (OR 1.43, 95% CI 1.11–1.84, *p *= 0.006), sleep quality (OR 1.19, 95% CI 1.00–1.41, *p *= 0.049), and adnexal surgery (OR 1.39, 95% CI 1.10–1.76, *p *= 0.006) remained associated with decreased AFC in women aged 31–36 years. The results of the multivariate analyses for AMH are presented in Table 4. After adjusting for other confounding factors, early age at menarche (OR 1.17, 95% CI 1.04–1.32, *p *= 0.007), alcohol drinking (OR 1.48, 95% CI 1.03–2.12, *p *= 0.033), and adnexal surgery (OR 1.52, 95% CI 1.13–2.05, *p *= 0.006) remained associated with AMH decline in women aged 20–30 years; and alcohol drinking (OR 1.52, 95% CI 1.02–2.26, *p *= 0.041) and adnexal surgery (OR 1.44, 95% CI 1.04–1.99, *p *= 0.027) remained associated with AMH decline in women aged 31–36 years.

**TABLE 3:** Multivariable logistic regression analysis of the risk factors associated with antral follicle count

Risk factors	31–36 years	
	OR (95% CI)	*P* value
Spontaneous abortion		
Yes	0.84 (0.58–1.22)	0.364
No	1	–
Moving or renovating		
Yes	0.63 (0.39–1.02)	0.061
No	1	–
Intense exercise		
Yes	1.43 (1.11–1.84)	0.006
No	1	–
Sleep quality		
Adnexal surgery	1.19 (1.00–1.41)	0.049
Yes	1.39 (1.10–1.76)	0.006
No	1	–

Abbreviations: CI, confidence interval; OR, odds ratio.

**TABLE 4:** Multivariable logistic regression analysis of the risk factors associated with anti‐Müllerian hormone

Risk factors	≤30 years		31–36 years	
	OR (95% CI)	*P* value	OR (95% CI)	*P* value
Age at menarche	0.85 (0.76–0.96)	0.007		
Alcohol drinking				
Yes	1.48 (1.03–2.12)	0.033	1.52 (1.02–2.26)	0.041
No	1	–		
Adnexal surgery				
Yes	1.52 (1.13–2.05)	0.006	1.44 (1.04–1.99)	0.027
No	1	–		

Abbreviations: CI, confidence interval; OR, odds ratio.

## 4 DISCUSSION

It is known that humans are facing a high risk of fertility decline. Factors affecting female fertility include age, female reproductive function status, genetic factors, and ovarian function. However, there is a relative lack of evaluation of specific factors affecting fertility in women of different ages, especially young women.^5^, ^12^ In this study, risk factors for ovarian reserve were analyzed in three age groups of women, matched for optimal age, suboptimal age, and advanced age.

Reproductive history and physiologic cycle are generally considered closely related to female fertility.^8^ Both univariate and multivariate analyses in this study showed that menarche age had a very significant negative impact on AMH in women aged 20–30 years, which was similar to the findings of previous studies.^6^, ^21^ Infertile women with early age at menarche may have a bigger follicular pool size and/or a faster follicular depletion rate, which in turn damages ovarian function.^21^ Our data suggest that menarche delay has a certain protective effect on female fertility, especially for women under 30.

Researchers have increasingly focused on the effects of new lifestyle, food intake, and environmental changes on fertility. Both univariate and multivariate analyses in this study showed that intense exercise and sleep quality have significant negative impacts on AFC in women aged 31–36 years, which was similar to a previous study.^12^ Excessive exercise may lower female fertility by reducing ovulation, damaging endometrial development, and inducing amenorrhea.^22^ Circadian dysrhythmia may interfere with fertility by altering the secretion of reproductive hormones, impairing reproductive outcomes and insulin resistance, and/or increasing inflammation.^23^ Our data suggested the importance of carefully considering the effects of intense exercise and sleep quality on female fertility, especially in women from 31 to 36 years of age. Alcohol drinking is generally considered to be related to female infertility. Univariate and multivariate analyses in this study showed significant negative associations between alcohol drinking and AMH in both the 20–30 and 31–36 year age groups, which was similar to previous reports.^9^ On the one hand, alcohol may lower female fertility through altering the endogenous hormone concentrations, hindering ovum maturation, and disturbing ovulation and early blastocyst development and implantation.^4^ In addition, toxicants present in alcohol such as ethyl carbamates may damage female fertility.^4^ Our data suggested that avoiding alcohol is important to female fertility protection, especially for women under 36 years of age. No association was found between smoking and AFC or AMH in our study, which was consistent with previous studies.^6^, ^7^ No association was found between coffee consumption and AFC or AMH in our study, which was consistent with a previous study.^9^

Pelvic and abdominal cavity surgeries have been widely perceived as negative factors because they may interfere with the blood supply of female reproductive organs and damage the normal microenvironment. Univariate and multivariate analysis confirmed that adnexal surgery had a significant negative impact on AFC and AMH in women aged 20–30 and 31–36 years, which was similar to previous studies.^24^ History of adnexal surgery may interfere with the blood supply of female ovaries and damage the normal microenvironment of antral follicle development. Our data suggested that women aged 20–36 years should be given a comprehensive assessment of ovarian function, a standardized intraoperative procedure, and a heightened awareness of ovarian protection before electing adnexal surgery. The postoperative fertility of patients with adnexal surgery should be considered, and individualized guidance is required for each patient. No adverse effects of uterine surgery on AFC or AMH were found, which was similar to a previous study.^25^

No significant correlations between any of the predictive risk factors and AFC or AMH levels were found in women aged 37 years and older. We speculated that women of this age group tend to have lower ovarian reserves already so other lifestyle insults or even adnexal surgery will not lower the ovarian reserves further. In addition, the small sample size may be part of the reason why external factors cannot affect the fertility of women from 37 years. Future studies should include a larger sample size of women aged 37 years and older.

Although female fertility has a certain ability to adapt to a variety of new lifestyles, environmental pollution, social behaviors, and medical factors, several age‐specific risk factors including adnexal surgery, intense exercise, poor sleep quality and drinking, should be taken seriously. Our study revealed that female fertility is more susceptible to external risk factors with age. For women over 37 years, the effects of external risk factors on fertility may be due to reduced physical function, such as lower ovarian reserve, and are not considered as consequential. These results provide more detailed data for protecting female fertility.

However, this study had a few limitations. It was a single‐center research and the study population was small, especially for the sub‐group of women aged 37 years or older, which might cause bias. Larger, multi‐center studies are needed to confirm the results of the current study. In addition, although significant associations were found between adnexal surgery and AFC and AMH levels, this work cannot demonstrate that it is an independent risk factor for AFC or AMH decline because it is difficult to rule out the effects of the diseases themselves and of different surgical skills on AFC and AMH levels.

## CONFLICTS OF INTEREST

The authors have no conflicts of interests.

## AUTHOR CONTRIBUTIONS

WH contributed to the conception and design of the study. DM, XL, YG, and FW were responsible for data collection and checking. YW, YY, and YL performed the data analysis, data interpretation, and manuscript drafting. All authors read and approved the final manuscript.
